# Real-world quality of life and sleep outcomes in patients treated with THC- and CBD-rich *Cannabis* oil: a cross-sectional study

**DOI:** 10.3389/fphar.2026.1862725

**Published:** 2026-07-22

**Authors:** Janniny Fernanda Lopes Mendes Figueiredo, Christian de Almeida Soares, Dario Alves de Oliveira, Fernando Ribeiro Cassiano, Veronica de Melo Sacramento, Afrânio Farias de Melo-Júnior, Elytania Veiga Menezes, Murilo Malveira Brandão, Vanessa de Andrade Royo

**Affiliations:** 1 Department of General Biology, Graduate Program in Biotechnology, State University of Montes Claros (Unimontes), Montes Claros, Minas Gerais, Brazil; 2 Center for Biological and Health Sciences (CCBS), School of Medicine, State University of Montes Claros (Unimontes), Montes Claros, Minas Gerais, Brazil; 3 Department of Exact Sciences, State University of Montes Claros (Unimontes), Montes Claros, Minas Gerais, Brazil

**Keywords:** cannabinoids, Cannabis, patient reported outcome measures, quality of life, sleep quality

## Abstract

The endocannabinoid system plays an important role in the modulation of pain, mood, sleep, and subjective wellbeing. Despite the growing clinical use of medicinal *Cannabis*, real-world data simultaneously evaluating quality of life and sleep-related outcomes in heterogeneous clinical populations remain limited. This study aimed to assess quality of life and sleep satisfaction in patients using medical *Cannabis* oil under supervised clinical follow-up. This cross-sectional observational study included patients treated with full-spectrum medical *Cannabis* oil rich in tetrahydrocannabinol (THC) and cannabidiol (CBD) in a real-world clinical setting. Quality of life was assessed using the World Health Organization Quality of Life–BREF (WHOQOL-BREF), and sleep quality was evaluated using the Pittsburgh Sleep Quality Index (PSQI). Sociodemographic, clinical, and treatment-related data were collected via an electronic questionnaire. Nonparametric analyses, Spearman correlation, and ordinal logistic regression models were performed. Seventy-one participants were included, predominantly female, with diverse clinical conditions. Participants reported generally favorable perceptions regarding quality of life and sleep satisfaction, with median scores concentrated in the higher response categories. Positive correlations were observed between the psychological domains of quality of life and sleep satisfaction. Higher *Cannabis* oil concentrations and longer treatment duration were associated with higher odds of better outcomes. In a real-world clinical context, supervised use of full-spectrum medical *Cannabis* oil was associated with favorable patient-reported perceptions of quality of life and sleep, consistent with perceived effectiveness. These findings highlight the need for longitudinal studies with pre-treatment baseline assessment.

## Introduction

*Cannabis sativa* L. [Cannabaceae] has a documented history of medicinal use spanning thousands of years across diverse cultural and geographic contexts, with traditional applications encompassing pain relief, mood modulation, sleep induction, and the management of inflammatory and neurological conditions (Zuardi, 2006; Russo, 2007). In Brazil, the therapeutic use of *Cannabis*-based products has been formally recognized within the national regulatory framework since 2015, with subsequent updates consolidating the supervised clinical use of cannabinoid-based botanical drugs for a broad spectrum of chronic conditions (ANVISA RDC No. 327/2019 and RDC No. 660/2022). The growing integration of *Cannabis* into contemporary clinical practice reflects both the long-standing ethnopharmacological tradition associated with this species and the expanding body of evidence supporting the pharmacological activity of its principal phytocannabinoid metabolites, tetrahydrocannabinol (THC) and cannabidiol (CBD) (Zuardi, 2006; Russo, 2007).

The endocannabinoid system plays a central role in the regulation of multiple physiological and behavioral processes, including nociception, mood, sleep, stress response, and subjective perception of wellbeing, primarily through activation of cannabinoid receptors CB1 and CB2, widely distributed in the central and peripheral nervous systems (Zou and Kumar, 2018; Lu and Mackie, 2016). This system acts as a fine homeostatic modulator, integrating neural, endocrine, and immunological signals (Zou and Kumar, 2018). Based on this biological framework, products derived from *C. sativa*, especially those containing cannabidiol (CBD) and tetrahydrocannabinol (THC), have been investigated as potential therapeutic interventions for various chronic clinical conditions, including chronic pain syndromes, with distinct and complementary mechanisms of action (Lu and Mackie, 2016; Cristino et al., 2020; Bort et al., 2024).

Over the past few decades, the use of *Cannabis* has expanded, driven by regulatory changes, greater acceptance in clinical practice, and growing appreciation of patient-centered outcomes (Zou and Kumar, 2018; Whiting et al., 2015). Systematic reviews and broad-scope studies suggest that cannabinoids may be associated with benefits in conditions such as chronic pain, anxiety, and sleep disorders; however, the magnitude and consistency of these effects vary substantially according to formulation, dose, clinical profile of patients, and context of use (Cristino et al., 2020; Pratt et al., 2019; Häuser et al., 2018). This variability reinforces the need for assessments that transcend exclusively biomedical outcomes and incorporate the subjective patient experience.

Sleep disorders are among the most frequently reported indications by individuals using *Cannabis*, especially among patients with painful, psychiatric, and neurological conditions (AminiLari et al., 2022; Babson et al., 2017; Amaral et al., 2023). Recent meta-analyses of randomized clinical trials indicate that cannabinoids may promote small to moderate improvements in subjective sleep quality compared to placebo (Suraev et al., 2020; Skevington et al., 2004). However, high methodological heterogeneity, short follow-up duration, and restricted sample selection limit the extrapolation of these findings to complex clinical and real-world practice settings.

In parallel, the assessment of health-related quality of life has become an essential component of contemporary clinical research, particularly in chronic diseases and long-term therapies (Skevington et al., 2004). Internationally validated instruments, such as the World Health Organization Quality of Life–BREF (WHOQOL-BREF) and the Pittsburgh Sleep Quality Index (PSQI), allow standardized measurement of physical, psychological, and social dimensions of patient experience, and are widely used in clinical and observational studies involving pharmacological and non-pharmacological interventions (Skevington et al., 2004; Buysse et al., 1989; Devlin and Brooks, 2017). The joint analysis of sleep quality and quality of life offers an integrated perspective on the functional and subjective impact of therapeutic interventions (Buysse et al., 1989).

Despite the growth of the literature on medical *Cannabis*, a relevant gap remains regarding the simultaneous evaluation of quality of life and sleep quality in heterogeneous clinical populations followed in real-world medical practice under supervised use of cannabinoid oils (Häuser et al., 2018; Suraev et al., 2020; Ware et al., 2010). Moreover, emerging evidence indicates that sociodemographic and clinical characteristics, such as age, self-reported racial profile, and treatment-related parameters, may influence the perception of patient-reported outcomes (Pratt et al., 2019; Häuser et al., 2018; Sat et al., 2015). In view of this context, the present study aimed to assess quality of life and sleep quality, using the Portuguese-validated versions of the WHOQOL-BREF and PSQI, in a cross-sectional sample of patients under medical follow-up, as well as to explore associations between comorbidities, sociodemographic characteristics, and therapeutic parameters in a real clinical practice context.

## Methods

### Study design and setting

This is a cross-sectional observational study conducted with patients on medical *Cannabis* oil treatment followed by physicians affiliated with the Therapeutic Association of Medical *Cannabis* Flor da Vida (Franca, São Paulo, Brazil). Participants were under continuous medical follow-up throughout the treatment, including clinical monitoring, individualized dose adjustments, medication concentration adequacy, laboratory review, and reassessment of concomitant therapies. The instruments were applied only after a defined period of oil use, with no pre-treatment baseline assessment.

### Investigational product

The product used consisted of full-spectrum *Cannabis* oil produced by the association itself, prepared from *C. sativa* extracts containing tetrahydrocannabinol (THC), cannabidiol (CBD), and other phytocannabinoid metabolites, diluted in medium-chain triglycerides (MCT). The concentrations used in the sample included formulations of 3% (3.75 mg/mL THC and 1.25 mg/mL CBD), 6% (7.50 mg/mL THC and 2.50 mg/mL CBD), 9% (15.00 mg/mL THC and 5.00 mg/mL CBD), and 18% (22.50 mg/mL THC and 7.50 mg/mL CBD), as well as other higher concentrations prescribed individually according to clinical need. All formulations maintained a fixed THC:CBD ratio of 3:1, with concentration as the variable parameter adjusted according to clinical indication. Dosing was individualized and determined by the attending physician based on clinical need, with gradual titration and periodic reassessment. Given the cross-sectional design of the study, adherence was not formally monitored; participants were assessed at a single point in time regarding their current use of the oil, treatment duration, and underlying clinical condition, without longitudinal follow-up. No biological samples were collected to objectively verify cannabinoid exposure or adherence. Prior *Cannabis* use history was not systematically assessed, which represents a limitation, as prior habituation to the endocannabinoid system in chronic users may influence treatment response.

### Plant material and cultivation

*Cannabis sativa* L. [Cannabaceae] plants used for oil production were cultivated by the Therapeutic Association Flor da Vida (Franca, São Paulo, Brazil) under a protected outdoor system (greenhouse structures). The cultivation site is located at an altitude of approximately 1,040 m above sea level, with a mean annual temperature of 21 °C, conditions considered favorable for phytocannabinoid expression. Plant material consisted exclusively of clonally propagated specimens derived from genetically characterized mother plants maintained by the association, ensuring genetic homogeneity and chemotypic consistency across production batches. Two chemovars were employed: Chemovar I (THC-predominant, <15% THC) and Chemovar III (CBD-predominant, <15% CBD), corresponding to the THC-rich and CBD-rich fractions used in the full-spectrum formulations. The propagation cycle, comprising cutting, rooting, and acclimatization stages, lasted approximately 60 days prior to field transplantation.

Soil management followed strictly biological and low-solubility mineral inputs, including dolomitic limestone, gypsum, and slow-release phosphate sources, supplemented with liquid biofertilizers throughout the growth cycle. Phytosanitary management adhered to Integrated Pest Management (IPM) principles, using exclusively organic and biological inputs; synthetic pesticides, fungicides, and systemic agrochemicals were strictly prohibited at all stages of the crop cycle. The field-to-harvest cycle comprised 90 days, followed by controlled drying for 10 days under regulated humidity and temperature conditions, in accordance with best practice guidelines for botanical drug characterization (Heinrich et al., 2022). The production and supply of medicinal *Cannabis* oil by the association is conducted within the Brazilian regulatory framework governing the use of cannabinoid-based products for therapeutic purposes, in accordance with ANVISA Resolution RDC No. 327/2019 and applicable complementary regulations. The association operates under the associative model recognized in Brazilian law for the collective cultivation and non-commercial supply of *Cannabis* derivatives to registered patients under medical supervision. All applicable phytosanitary and quality requirements were observed throughout the cultivation and processing chain.

### Extraction process

The full-spectrum *Cannabis* oil was produced through a standardized multi-step process developed in technical collaboration with UNESP-Araraquara and conducted in accordance with Good Manufacturing Practices (GMP) for phytotherapeutic products (ANVISA Normative Instruction No. 130/2022). Briefly, dried female inflorescences of *C. sativa L* (10 kg per batch) were subjected to turbo-extraction in anhydrous ethanol (3,450 rpm, 10 min) at a plant-to-solvent ratio of 1:12 (w/v), followed by maceration in stainless steel tanks (120 L) at a controlled room temperature of 16 °C–18 °C. The resulting ethanolic extract was subsequently subjected to winterization at −20 °C for 48 h, followed by vacuum filtration through a quantitative filter paper (Büchner funnel) to remove waxes, particulate matter, and lipophilic metabolites. Solvent removal was performed using a rotary evaporator (IKA RV 3 ECO) at 50 °C and −25 mmHg until complete ethanol evaporation. The concentrated crude extract was then decarboxylated to convert cannabinoid acid forms (THCA, CBDA) into their pharmacologically active neutral forms (THC, CBD), either by open-system water bath heating (85 °C–90 °C, approximately 12 h, with mechanical stirring) or by closed-system rotary evaporation (90 °C, 8 h, under vacuum). Decarboxylation completion was verified analytically by a responsible pharmacist prior to batch release. The resulting decarboxylated extract was diluted in medium-chain triglycerides (MCT) to yield the final formulations used in clinical practice.

### Phytochemical characterization

Batch-specific phytocannabinoid profiling was performed using HPLC-DAD (POP LTA MET-402; CIATox, Campinas, Brazil) on a representative production batch (Lot 220825) collected during the study data collection period (August 2025). Analysis of the crude extract prior to MCT dilution showed a THC content of 44.9% (449 mg/g) and a CBD content of 15.6% (156 mg/g), corresponding to a THC:CBD ratio of 3:1. Following standardized dilution in MCT, the final formulations yielded the concentrations described above (3.75/1.25 mg/mL, 7.50/2.50 mg/mL, 15.00/5.00 mg/mL, and 22.50/7.50 mg/mL THC/CBD for the 3%, 6%, 9%, and 18% formulations, respectively). Acid forms (THCA, CBDA) and minor cannabinoids (CBDV, CBG, CBN, delta-8-THC, CBGA, CBC) were below the limit of quantification (<LIQ; 1.6% m/m) across the full cannabinoid panel (Heinrich et al., 2022).

Reference standard specifications used in the HPLC-DAD analysis were not available for disclosure at the time of manuscript preparation; the analytical laboratory (CIATox) follows accredited internal procedures for cannabinoid quantification in accordance with applicable regulatory standards.

### Quality control

Batch-specific quality control analyses were conducted by Suprema Analítica (Franca, São Paulo, Brazil) on the same production batch (Lot 220825), collected during the study data collection period. Mycotoxin screening (aflatoxins B1, B2, G1, G2) was performed according to ISO 16050:2003, with all results below the limit of quantification (<0.50 μg/L). Heavy metal analysis (arsenic, cadmium, lead, mercury) was conducted following internal procedure IT 097, with all analytes below the respective detection limits (<0.0050 mg/L, <0.0010 mg/L, <0.0010 mg/L, and <0.0001 mg/L, respectively). Microbiological safety was assessed for *Escherichia coli*, *Salmonella* spp., *Pseudomonas* spp., *Staphylococcus aureus*, sulfite-reducing clostridia, *Clostridium perfringens*, thermotolerant coliforms, and yeasts and molds, with all results below the respective limits of quantification. These results confirm the absence of relevant microbiological, mycotoxin, and heavy metal contamination in the analyzed batch, consistent with safety standards for botanical medicinal products and in accordance with ConPhyMP reporting guidelines (Heinrich et al., 2022).

### Inclusion criteria

Eligible patients were those actively using *Cannabis* oil with a formal medical prescription, regularly followed by association physicians, regardless of the underlying comorbidity. Adult and pediatric patients were included, provided they were under active clinical follow-up and had responded completely to the questionnaire during the data collection period. For minors, written informed consent (TCLE) was obtained from parents or legal guardians, and written informed assent (TALE) was obtained from participants who demonstrated sufficient capacity to understand and agree to participation.

### Data collection

Data collection occurred between May and September 2025, via a self-administered electronic form (Google Forms), sent by e-mail to eligible patients. A total of 689 patients were contacted and invited to participate in the study, and 71 complete valid responses were obtained for analysis. The questionnaire included sociodemographic, clinical, and therapeutic variables, as well as standardized instruments for assessing quality of life and sleep quality.

### Assessment instruments

Quality of life was assessed using the validated Brazilian version of the World Health Organization Quality of Life–BREF (WHOQOL-BREF), comprising 26 questions distributed across physical, psychological, social relations, and environment domains, plus two general questions on global perception of quality of life and health satisfaction. Sleep quality was measured using the validated Brazilian version of the Pittsburgh Sleep Quality Index (PSQI), which assesses subjective and objective sleep components over the 4 weeks prior to administration.

### Data processing and variable grouping

Due to the observational and real-world clinical nature of the study, several variables collected through the self-administered questionnaire showed heterogeneity in response format, level of detail, and possibility of multiple selections. Therefore, a systematic data treatment and categorization strategy was adopted prior to analyses, to ensure analytical consistency, statistical stability, and clinical interpretability of results.

Age was originally collected as a continuous variable and, for analytical purposes, categorized according to the World Health Organization (WHO) life course classification, including the categories: child, adolescent, young adult, adult, middle-aged, early elderly, and advanced elderly. This approach allowed clinically meaningful comparisons between age groups, while reducing dispersion and low frequencies in isolated categories.

Clinical conditions were reported in free-text format, frequently involving multiple diagnoses per participant. Reported pathologies were grouped into pre-defined clinical categories, including psychiatric/emotional conditions, neurological or neurodevelopmental conditions, chronic pain/musculoskeletal conditions, autoimmune or systemic diseases, oncological conditions, gynecological conditions, post-surgical conditions, and other less frequent clinical conditions. Participants could be classified into more than one comorbidity group, reflecting the clinical complexity observed in real-world practice.

Variables related to treatment with *Cannabis* oil, such as treatment duration, time to reach the ideal dose, oil concentration used, and concomitant use of other medications, were originally reported in varied formats. For analytical purposes, these variables were recoded into ordinal categories, aiming to increase comparability among participants and ensure adequate sample size in each stratum. Adverse effects, due to the high variability and low individual frequency of specific symptoms, were grouped dichotomously (presence or absence of any adverse event).

Responses to the WHOQOL-BREF and PSQI instruments were analyzed respecting the ordinal nature of Likert-type scales. For a limited number of items in which the electronic form allowed multiple responses per participant, the highest recorded score was considered for analysis. This criterion was adopted to maintain consistency in ordinal coding and to capture the most favorable scenario explicitly indicated by the respondent. It is acknowledged that this approach may have introduced a degree of upward bias in individual item scores; accordingly, future applications of the instrument should be configured to prevent multiple selections per item. It is important to note that the instruments include items with both positive and negative directionality. In the present dataset, most items affected by multiple responses were negatively oriented, that is, higher original scores corresponded to less favorable responses for the participant. For these items, retaining the highest original score before reverse coding represented a conservative approach, preserving the least favorable response among those selected by the participant, rather than a procedure that systematically inflated favorable outcomes. The statistically significant results were reviewed considering the occurrence of multiple responses, and no relevant change in scores or in the overall interpretation of the findings was observed. This is because multiple responses occurred in only a small proportion of the dataset and did not materially affect the global analytical pattern. Future applications of the questionnaire should be configured to allow only one response per item to avoid ambiguity at the data entry stage. Responses marked as ‘prefer not to answer’ were treated as non-informative and excluded from inferential analyses. Missing data resulted in variation in sample size across analyses, and pairwise deletion was applied depending on the variable analyzed. The grouping and categorization process was guided by the preservation of clinical relevance, reduction of low-frequency categories, and adequacy for application of nonparametric tests and ordinal regression models, considering the sample size and actual distribution of responses.

### Ethical aspects

The study was conducted in accordance with the ethical principles of the Declaration of Helsinki. The protocol was submitted to the Ethics Commission–Plataforma Brasil and approved by the CEP/Unimontes, according to consolidated opinion no. 7.390.935, in compliance with CNS Resolutions no. 466/12, no. 510/16, and their complementary regulations. The study included pediatric and adolescent participants (children aged <10 years, n = 4; adolescents aged 10–18 years, n = 4). For all minor participants, written informed consent (Informed Consent Form–ICF) was obtained from parents or legal guardians prior to enrollment. Written informed assent (Informed Assent Form–IAF) was additionally obtained from participants who demonstrated sufficient capacity to understand and agree to participation, in accordance with the ethical standards of the Research Ethics Committee and CNS Resolution No. 466/12. Data from all minor participants were handled with the same anonymization procedures applied to adult participants. All participants provided formal authorization through signing the ICF and IAF, and data were analyzed anonymously, ensuring privacy and confidentiality.

### Statistical analysis

Statistical analyses were conducted using IBM SPSS Statistics version 20. Initially, descriptive analysis of sociodemographic, clinical, and therapeutic variables was performed, with calculation of absolute and relative frequencies, means, standard deviations, medians, and interquartile intervals. Normality of the main outcomes was assessed by the Kolmogorov–Smirnov and Shapiro–Wilk tests, which indicated non-normal distribution, consistent with the ordinal nature of Likert scales.

Comparisons between two independent groups were performed using the Mann–Whitney test. For comparisons involving three or more independent groups, one-way ANOVA was applied as a complementary and comparative analysis, retained because it presented a lower residual error under the specific conditions of the sample. The inferential interpretation of the ANOVA was conducted with caution, given that the test assumes approximately normal distribution and homogeneity of variances, assumptions that may be challenged by ordinal Likert-type scales. Accordingly, medians and interquartile ranges were prioritized in the interpretation of group differences. Bivariate associations between variables were assessed using the Spearman correlation coefficient (ρ), after standardization and rescaling of scales to ensure uniform interpretative direction. Additionally, ordinal logistic regression models were fitted to identify factors associated with the outcomes “quality of life assessment” and “sleep satisfaction”. Given the sample size (n = 71) and the presence of sparse categories in some predictors, these models are considered exploratory in nature. Some predictors with very low cell frequencies produced unstable estimates, including extreme coefficients and non-estimable standard errors, a phenomenon consistent with quasi-complete separation. Results from these specific parameters should be interpreted with caution and not used for direct clinical inference. A significance level of 5% (*α* = 0.05) was adopted for all analyses.

## Results

A total of 71 participants were included in the final analysis. Female predominance was observed (71.8%), with heterogeneous age distribution and higher concentration in the middle-aged (31.0%) and adult (26.8%) categories. Age ranged from 4 to 89 years, with a mean of 48.3 ± 21.2 years, a median of 51.0 years, and an interquartile range of 36.0–60.5 years. The majority of participants self-identified as white (62.0%), followed by brown (31.0%). Among the clinical groups, psychiatric/emotional conditions (36.6%), neurological/neurodevelopmental conditions (21.1%), and chronic musculoskeletal pain (11.3%) stood out. Regarding medical *Cannabis* treatment, the most frequently used oil concentration was 6% (43.7%), followed by 9% (19.7%) and 3% (14.1%). Regarding therapeutic management, 50.7% reported concomitant use of other medications, 42.3% reported not using additional drugs, and 7.0% did not respond to the question. Among the 32.4% who reported adverse events, the most frequent were xerostomia (4.2%), agitation, somnolence, and vertigo (2.8% each), as shown in Table 1.

**TABLE 1:** Sociodemographic and clinical characteristics of participants (n = 71).

Category	Subcategory	(n)	%
Age (Years)	Mean ± SD	48.3 ± 21.2	—
	Median (IQR)	51.0 (36.0–60.5)	—
	Range	4–89	—
Age group	Child	4	5.6
	Adolescent	4	5.6
	Young adult	2	2.8
	Adult	19	26.8
	Middle-aged	22	31.0
	Early elderly	13	18.3
	Advanced elderly	7	9.9
Sex	Female	51	71.8
	Male	20	28.2
Race	White	44	62.0
	Brown	22	31.0
	Other	5	7.0
Gender identity	Cisgender woman	48	67.6
	Cisgender man	18	25.4
	Prefer not to answer	5	7.0
Sexual orientation	Heterosexual	64	90.1
	Bisexual	1	1.4
	Pansexual	1	1.4
	Prefer not to answer	5	7.0
Treatment duration	1–3 months	12	16.9
	3–6 months	5	7.0
	6–9 months	7	9.9
	9–12 months	8	11.3
	1–1.5 years	8	11.3
	1.5 years	12	16.9
	2–3 years	4	5.6
	>3 years	15	21.1
Time to ideal dose	1–3 months	32	45.1
	3–6 months	16	22.5
	6–9 months	9	12.7
	9–12 months	8	11.3
	1–1.5 years	2	2.8
	1.5–2 years	2	2.8
	>3 years	2	2.8
Cannabis oil concentration	6%	31	43.7
	9%	14	19.7
	3%	10	14.1
	18%	9	12.7
	Other	7	9.9
Adverse effects	No	48	67.6
	Yes	23	32.4
Concomitant medications	Yes	36	50.7
	No	30	42.3
	No response	5	7.0
Clinical conditions	Psychiatric/Emotional	26	36.6
	Neurological/Neurodevelopmental	15	21.1
	Chronic musculoskeletal pain	8	11.3
	Other conditions/Associations	8	11.3
	Neurological + psychiatric	3	4.2
	Chronic pain + psychiatric	3	4.2
	Gynecological	2	2.8
	Oncological	2	2.8
	Psychiatric + chronic pain	2	2.8
	Other	2	2.8

The global assessment of quality of life had a mean of 3.13 (SD = 1.40), median of 4, and mode corresponding to the “satisfied” category. Sleep satisfaction had a mean of 3.23 (SD = 1.18), also with a median of 4, indicating concentration of responses in the higher levels of the Likert scale, as shown in Table 2. The response distribution showed positive skewness, with predominance of scores 4 and 5. Normality tests evidenced non-normal behavior for quality of life (Kolmogorov–Smirnov = 0.282; *p* < 0.001; Shapiro–Wilk = 0.844; *p* < 0.001) and sleep satisfaction (Kolmogorov–Smirnov = 0.257; *p* < 0.001; Shapiro–Wilk = 0.878; *p* < 0.001), as shown in Table 3, justifying the use of nonparametric analyses.

**TABLE 2:** Descriptive comparison of outcomes.

Analyzed indicator	Sample (N)	Mean	Standard deviation	Central tendency (mode)
Quality of life	71	3.13	1.40	4 (satisfied)
Sleep satisfaction	70	3.23	1.18	4 (satisfied)

**TABLE 3:** Normality tests for quality of life and sleep satisfaction.

Outcome	Kolmogorov–Smirnov statistic	DF	*p*	Shapiro–Wilk statistic	DF	*p*
Quality of life assessment	0.282	71	<0.001	0.844	71	<0.001
Sleep satisfaction	0.257	70	<0.001	0.878	70	<0.001

DF, degrees of freedom.

In comparative analyses between independent groups, no statistically significant differences were observed in quality of life assessment or sleep satisfaction across any of the sociodemographic and clinical variables examined, including sex, race, age group, treatment duration, oil concentration, and clinical condition groups (all p > 0.05). Age group comparisons approached but did not reach statistical significance for quality of life (p = 0.061). These analyses are presented in Tables 4, 5 as exploratory and descriptive comparisons, intended to characterize the sample rather than to establish group differences.

**TABLE 4:** Comparative analysis of quality of life across sociodemographic and clinical variables.

Variable	Group	Mean ± SD	Median	*p*-value	Test
Quality of life					
Sex	Female	3.04 ± 0.204	4,0	0.539	Mann-Whitney U
	Male	3.30 ± 0.30	4,0		
Adverse effects	Yes	3.09 ± 0.30	4,0	0.705	Mann-Whitney U
	No	3.13 ± 0.214	4,0		
Race	Pardo	3.19 ± 0.306	4.0	0.906	One-way ANOVA
	White	3.07 ± 0.221	4.0		
	Other	3.20 ± 0.490	4.0		
Age	Child	3.25 ± 1.5	4.0	0.061	One-way ANOVA
	Adolescent	2.75 ± 1.5	3.0		
	Young adult	5.0 ± 0	5.0		
	Adult	3.26 ± 1.327	3.5		
	Middle-aged	2.41 ± 1.368	2.0		
	Early older adult	3.69 ± 1.251	4.0		
	Advanced older adult	3.57 ± 1.272	4.0		
Treatment duration	1–3 months	2.50 ± 1.080	2.0	0.061	One-way ANOVA
	3–6 months	2.20 ± 1.640	1.0		
	6–9 months	3.43 ± 1.270	4.0		
	9–12 months	2.13 ± 1.120	2.0		
	1–1.5 years	3.50 ± 1.310	4.0		
	1.5 years	3.55 ± 1.510	4.0		
	2–3 years	4.25 ± 0.500	4.0		
	More than 3 years	3.47 ± 1.510	4.0		
Oil concentration	3%	3.30 ± 1.250	4.0	0.741	One-way ANOVA
	6%	3.03 ± 1.300	3.5		
	9%	3.29 ± 1.490	4.0		
	18%	3.44 ± 4.670	4.0		
	Other	2.57 ± 1.720	2.0		
Clinical condition groups	Chronic pain/Musculoskeletal	3.25 ± 1.282	3.5	0.098	One-way ANOVA
	Chronic pain/Musculoskeletal; psychiatric/Emotional	1.50 ± 0.707	1.5		
	Gynecological	1.50 ± 0.707	1.5		
	Neurological/Neurodevelopmental	3.00 ± 1.000	4.0		
	Neurological/Neurodevelopmental; psychiatric/Emotional	4.50 ± 0.707	3.0		
	Oncological	3.27 ± 1.185	4.5		
	Psychiatric/Emotional	3.5 ± 0.707	3.3		
	Psychiatric/Emotional; chronic pain/Musculoskeletal	3.5 ± 1.195	3.5		
	Other conditions/Associations	2.00 ± 1.414	4.0		
	Others	3.23 ± 1.182	2.0		

**TABLE 5:** Comparative analysis of sleep satisfaction across sociodemographic and clinical variables.

Variable	Group	Mean ± SD	Median	*p*-value	Test
Sleep satisfaction					
Sex	Female	3.10 ± 0.167	3.0	0.155	Mann–Whitney U
	Male	3.55 ± 0.256	4.0		
Adverse effects	Yes	3.43 ± 0.250	4.0	0.304	Mann–Whitney U
	No	3.13 ± 0.171	3.0		
Use of other medications	Yes	3.36 ± 1.099	3.5	0.547	Mann–Whitney U
	No	3.07 ± 1.361	4.0		
Race	Mixed (pardo)	2.90 ± 0.266	3.0	0.295	One-way ANOVA
	White	3.34 ± 0.175	4.0		
	Other	3.60 ± 0.510	4.0		
Age	Child	4.25 ± 0.957	4.5	0.132	One-way ANOVA
	Adolescent	3.50 ± 0.577	3.5		
	Young adult	4.00 ± 1.414	4.0		
	Adult	3.28 ± 1.127	3.5		
	Middle-aged	2.73 ± 1.279	3.0		
	Early older adult	3.62 ± 0.870	4.0		
	Advanced older adult	3.00 ± 1.414	3.0		
Treatment duration	1–3 months	2.92 ± 1.24	3.5	0.929	One-way ANOVA
	3–6 months	2.8 ± 1.643	2.0		
	6–9 months	3.43 ± 1.134	4.0		
	9–12 months	3.50 ± 0.926	3.5		
	1–1.5 years	3.27 ± 1.22	4.0		
	1.5–2 years	3.25 ± 1.28	3.5		
	2–3 years	3.00 ± 1.414	3.5		
	More than 3 years	3.40 ± 1.121	4.0		
Oil concentration	3%	3.60 ± 1.265	4.0	0.178	One-way ANOVA
	6%	3.00 ± 1.232	3.0		
	9%	3.29 ± 0.914	3.5		
	18%	3.89 ± 1.05	3.5		
Clinical condition groups	Chronic pain/Musculoskeletal	3.25 ± 1.282	3.5	0.098	One-way ANOVA
	Chronic pain/Musculoskeletal; psychiatric/Emotional	1.50 ± 0.707	1.5		
	Gynecological	1.50 ± 0.707	1.5		
	Neurological/Neurodevelopmental	3.00 ± 1.000	4.0		
	Neurological/Neurodevelopmental; psychiatric/Emotional	4.50 ± 0.707	3.0		
	Oncological	3.27 ± 1.185	4.5		
	Psychiatric/Emotional	3.5 ± 0.707	3.3		
	Psychiatric/Emotional; chronic pain/Musculoskeletal	3.5 ± 1.195	3.5		
	Other conditions/Associations	2.00 ± 1.414	4.0		
	Others	3.23 ± 1.182	2.0		​

In the comparison between comorbidity groups, discrete variations were observed in central tendency measures of quality of life. Participants with neurological/neurodevelopmental conditions had a mean of 3.00 ± 1.00 and median of 4.0, while the psychiatric/emotional group had an approximate mean of 3.50 ± 0.70 and median of 3.3. Individuals with chronic musculoskeletal pain had a mean of 3.25 ± 1.28 and median of 3.5, with greater interquartile range. Oncological groups showed higher central values (4.00 ± 1.41; median = 4.5), while participants classified under other clinical conditions had a mean of 2.00 ± 1.41 and median of 4.0. Despite these descriptive differences, there was no overall statistical significance between groups (Kruskal–Wallis, *p* = 0.098), as shown in Table 5.

In bivariate association analysis by Spearman correlation, a strong correlation was observed between global quality of life assessment and health satisfaction (ρ = 0.841; p < 0.001). The remaining correlations involving the quality of life assessment variable showed weak to moderate magnitudes. A positive correlation was also observed between the psychological domains of the WHOQOL-BREF and sleep satisfaction, with coefficients ranging from ρ = 0.20 to ρ = 0.39, characterizing a weak to moderate association between subjective perception of psychological wellbeing and sleep quality. For the sleep satisfaction variable, the highest correlation coefficient was observed with the sleep satisfaction assessment itself (ρ = 0.685; p < 0.001). The second highest correlations were identified with the variables sufficient energy for daily activities (ρ = 0.472; p < 0.001) and problems falling asleep/difficulty initiating sleep (ρ = 0.458; p < 0.001). The correlation between quality of life assessment and sleep satisfaction was of weak to moderate magnitude (ρ = 0.332; p < 0.001).

In the multivariate ordinal logistic regression model for quality of life, adult participants showed a lower probability of higher outcome levels compared to the reference group (advanced elderly) (β = −4.403; p = 0.043; 95% CI −8.676 to −0.131). Regarding racial self-declaration, brown participants (β = −8.80; p = 0.001) and white participants (β = −10.11; p ≤ 0.001) showed lower probabilities of higher quality of life levels. Patients using the oil for 3–12 months and 1.5–2 years had a lower probability (β < 0) of reporting higher satisfaction levels compared to those using the medication for more than 3 years. The variable sex was not significant (p > 0.05). Use of 9% oil concentration was associated with a higher probability of high quality of life levels (β = 8.03; p = 0.002), as shown in Table 6.

**TABLE 6:** Ordinal logistic regression model for quality of life.

95% CI Upper	Variable	β	Std. error	Wald (W)	df	p-value	95% Confidence interval	
							Lower	Upper
Age	Child	0.317	2.945	0.012	1	0.914	−5.455	6.089
	Adolescent	−4.934	2.518	3.840	1	0.050	−9.869	0.001
	Young adult	30.694	9,894.424	0.000	1	0.998	−19362.02	19423.408
	Adult	−4.403	2.180	4.081	1	0.043	−8.676	−0.131
	Middle-aged	−3.908	2.456	2.533	1	0.111	−8.721	0.905
	Early elderly	3.243	2.118	2.345	1	0.126	−0.908	7.394
	Advanced elderly	0.000	NE	NE	0	NE	NE	NE
Race	Brown	−8.802	2.602	11.440	1	0.001	−13.903	−3.702
	White	−10.106	2.820	12.846	1	0.000	−15.632	−4.580
	Other	0.000	NE	NE	0	NE	NE	NE
Condition groups	Chronic/Musculoskeletal pain	−0.077	3.011	0.001	1	0.980	−5.978	5.825
	Chronic pain/Musculoskeletal; psychiatric/Emotional	−3.127	3.463	0.815	1	0.367	−9.914	3.661
	Gynecological	8.382	4.704	3.174	1	0.075	−0.839	17.602
	Neurological/Neurodevelopmental	4.222	3.421	1.523	1	0.217	−2.484	10.927
	Neurological/Neurodevelopmental; psychiatric/Emotional	9.048	5.721	2.501	1	0.114	−2.165	20.260
	Oncological	6.889	4.161	2.742	1	0.098	−1.266	15.044
	Psychiatric/Emotional	6.665	3.338	3.987	1	0.046	0.123	13.207
	Psychiatric/Emotional; chronic pain/Musculoskeletal	9.718	8.152	1.421	1	0.233	−6.260	25.696
	Other conditions/Associations	−2.700	3.089	0.764	1	0.382	−8.754	3.355
	Others	0.000	NE	NE	0	NE	NE	NE
Treatment duration	0–3 months	6.147	6.538	0.884	1	0.347	−6.668	18.962
	1–3 months	−9.484	2.709	12.254	1	0.000	−14.795	−4.174
	3–6 months	−14.759	3.615	16.665	1	0.000	−21.845	−7.673
	6–9 months	−3.892	2.286	2.899	1	0.089	−8.372	0.588
	9–12 months	−12.751	3.567	12.780	1	0.000	−19.742	−5.760
	1–1.5 years	−3.619	2.316	2.442	1	0.118	−8.159	0.921
	1.5–2 years	1.954	2.253	0.752	1	0.386	−2.461	6.368
	2–3 years	0.877	3.294	0.071	1	0.790	−5.580	7.334
	>3 years	0.000	NE	NE	0	NE	NE	NE
Oil concentration	3%	1.568	2.402	0.426	1	0.514	−3.141	6.276
	6%	3.655	2.250	2.639	1	0.104	−0.755	8.065
	9%	8.032	2.548	9.934	1	0.002	3.037	13.027
	18%	0.135	2.585	0.003	1	0.958	−4.932	5.201
	Other	0.000	NE	NE	0	NE	NE	NE

Abbreviations: β = regression coefficient; Std. Error = standard error; df = degrees of freedom; NE, not estimable. Models are exploratory given the sample size. Parameters marked NE, are non-estimable due to quasi-complete separation resulting from sparse category frequencies.

For sleep quality, participants who reported absence of adverse effects showed a lower probability of high outcome levels compared to those who reported adverse events (β = −2.67; p = 0.008; 95% CI −4.654 to −0.683). Regarding time to reach ideal dosage, intervals of 1.5–2 years (β = −9.91; p = 0.022), 3–6 months (β = −7.29; p = 0.042), and 6–9 months (β = −8.40; p = 0.034) showed lower probabilities of high sleep satisfaction levels compared to the reference group (>3 years). Regarding treatment duration, no statistically significant associations were observed with sleep satisfaction in the regression model (Table 7). Some categories produced unstable estimates due to sparse cell frequencies, consistent with quasi-complete separation, and should be interpreted with caution. Racial self-declaration maintained a significant association, with brown participants (β = −7.03; p = 0.003) and white participants (β = −6.36; p = 0.006) showing a lower probability of higher outcome levels. No significant associations were observed for sex or clinical condition group (p > 0.05), as shown in Table 7.

**TABLE 7:** Ordinal logistic regression model for sleep satisfaction.

95% CI Upper	Variable	β	Std. error	Wald (W)	df	p-value	95% Confidence interval	
							Lower	Upper
Race	Brown	−7.026	2.401	8.565	1	0.003	−11.731	−2.320
	White	−6.363	2.306	7.614	1	0.006	−10.883	−1.843
	Other	0.000	NE	NE	0	NE	NE	NE
Treatment duration	0–3 months	0.624	1.288	0.235	1	0.628	−1.900	3.148
	1–3 months	−2.016	1.644	1.503	1	0.220	−5.239	1.207
	3–6 months	0.415	1.528	0.074	1	0.786	−2.580	3.411
	6–9 months	1.528	1.835	0.693	1	0.405	−2.068	5.124
	9–12 months	0.310	1.658	0.035	1	0.852	−2.940	3.560
	1–1.5 years	1.677	1.372	1.494	1	0.222	−1.012	4.365
	1.5–2 years	−0.158	1.713	0.008	1	0.927	−3.516	3.200
	2–3 years	0.000	NE	NE	0	NE	NE	NE
	>3 years	0.624	1.288	0.235	1	0.628	−1.900	3.148
Time to reach ideal dose	1–3 months	−6.695	3.608	3.444	1	0.063	−13.766	0.375
	3–6 months	−7.294	3.585	4.139	1	0.042	−14.321	−0.267
	6–9 months	−8.403	3.973	4.473	1	0.034	−16.190	−0.616
	9–12 months	−7.717	3.785	4.158	1	0.041	−15.135	−0.300
	1–1.5 years	7.406	0.000	NE	1	NE	7.406	7.406
	1.5–2 years	−9.909	4.331	5.235	1	0.022	−18.397	−1.420
	>3 years	0.000	NE	NE	0	NE	NE	NE
Adverse effects	No	−2.668	1.013	6.941	1	0.008	−4.654	−0.683
	Yes	0.000	NE	NE	0	NE	NE	NE

Abbreviations: β = regression coefficient; Std. Error = standard error; df = degrees of freedom; NE, not estimable. Models are exploratory given the sample size. Parameters marked NE, are non-estimable due to quasi-complete separation resulting from sparse category frequencies. The identical coefficients observed for the ‘0–3 months’ and ‘>3 years’ treatment duration categories reflect quasi-complete separation in this variable, resulting from the sparse distribution of observations across categories relative to the sample size. These estimates are not independently interpretable.

## Discussion

The present study evaluated, in a real clinical practice context, quality of life and sleep satisfaction in patients under supervised use of *Cannabis* oil rich in THC and CBD, using validated instruments and patient-reported outcomes relevant for capturing functional and subjective impact in heterogeneous chronic conditions. In the present study, the predominance of high scores for both outcomes suggests a globally favorable self-reported perception of treatment. Consistent with prior evidence, observational studies have described favorable patient-reported outcomes in medical *Cannabis* cohort studies (Arkell et al., 2023; Habib et al., 2021; Aviram et al., 2021; Cahill et al., 2021). Together, these findings reinforce the importance of evaluating patient-perceived benefits, especially in complex interventions where the clinical effect may involve multiple domains (pain, sleep, mood, functioning, and wellbeing) and not just an isolated biomedical marker (Zou and Kumar, 2018; Lu and Mackie, 2016; Cristino et al., 2020; AminiLari et al., 2022; Suraev et al., 2020; Skevington et al., 2004).

Regarding the age–quality of life axis, although comparisons between age groups did not reach conventional statistical significance in bivariate analyses in the present study, the direction of effects observed in the ordinal regression model suggests that adults had a lower probability of high quality of life levels compared to more advanced elderly, which requires clinical and contextual interpretation. In moderate observational samples, the absence of significance does not necessarily exclude clinical relevance, and it is more appropriate to jointly interpret the pattern of effect, plausibility, and coherence with the literature, especially when the outcome is self-reported and multidimensional (Skevington et al., 2004; Devlin and Brooks, 2017). Synthesis studies on pain and sleep with cannabinoids also highlight important heterogeneity of response according to population and study design, which may influence subgroup comparisons by age (Häuser et al., 2018; AminiLari et al., 2022; Suraev et al., 2020; Skevington et al., 2004). Moreover, expectations and adaptation to illness vary throughout the life course and may influence how therapeutic gains are translated into global assessments of wellbeing and satisfaction (Häuser et al., 2018; Skevington et al., 2004; Winiger et al., 2021). Notably, age has been identified as a moderator of the relationship between CBD concentration and sleep outcomes, with older adults showing a stronger association between higher CBD content and improved sleep efficiency and duration (Winiger et al., 2021).

At the extremes of age, especially in pediatric and adolescent populations with neurological and neurodevelopmental conditions, interpretation must be even more cautious due to diagnostic heterogeneity and different clinical trajectories. Proof-of-concept randomized trials in autism spectrum disorder suggest possible benefit in some clinical and sleep/behavior-associated outcomes, although methodological limitations and the need for standardization of formulations and outcomes persist (Aran et al., 2021). Additionally, reviews on cannabinoids and sleep describe variability of response according to clinical profile, cannabinoid metabolite used, and context of use, reinforcing the need to avoid simplistic extrapolations between populations and products (Suraev et al., 2020; Gates et al., 2014). Thus, the findings of the present study, while encouraging, are best interpreted as a signal of perceived effectiveness in clinical practice, and not as definitive evidence of comparative efficacy by age group.

A distinctive aspect of the present study is the use of a full-spectrum (chemotype I) oil with THC predominance, in contrast to part of the literature that favors CBD-rich formulations or standardized preparations. Systematic reviews and evidence overviews describe that effects and tolerability vary substantially according to formulation, dose, THC:CBD ratio, and patient selection, which may explain discrepant results and hinder direct comparability (Pratt et al., 2019; Häuser et al., 2018; AminiLari et al., 2022). In this context, the association observed in the present study between the 9% concentration and a higher probability of high quality of life levels should be treated with caution, since cross-sectional designs do not allow inferring causality or dose-response relationships, and concentrations may function as indirect markers of therapeutic phase, titration, clinical indication, or severity profile (AminiLari et al., 2022; Suraev et al., 2020; Skevington et al., 2004; Arkell et al., 2023). In other words, the concentration may reflect a “balance point” achieved by clinical adjustment over follow-up, rather than representing an isolated and linear pharmacological effect of the cannabinoid content.

The phytochemical composition is particularly relevant for interpreting the results observed with complex extracts such as the one used in this study. The “entourage effect” hypothesis proposes synergies between phytocannabinoids and terpenoids that could modulate efficacy, tolerability, and subjective experience, and is frequently invoked to explain differences between full-spectrum extracts and isolated cannabinoids (Russo, 2011). Although it remains a controversial and difficult-to-conclusively demonstrate topic in humans, this framework helps contextualize why different products may produce different response and adverse event profiles, especially in patient-centered outcomes such as sleep and quality of life (Arkell et al., 2023; Russo, 2011). Furthermore, studies and syntheses focused on prolonged use suggest that changes in composition and titration patterns over time may accompany variations in quality of life and analgesic consumption, reinforcing the need for product characterization in real clinical studies (Cahill et al., 2021; Bialas et al., 2022).

A key finding was the relationship between sleep and wellbeing as an integrating axis, evidenced by the positive correlation between psychological domains of the WHOQOL-BREF and sleep satisfaction. Consistent with prior evidence, systematic reviews and scoping reviews on cannabinoids and sleep indicate that subjective improvements are frequent but inconsistent, varying substantially according to population, metabolite, dose, ratio of cannabinoids, and route of administration (AminiLari et al., 2022; Babson et al., 2017; Amaral et al., 2023; Suraev et al., 2020; Gates et al., 2014). The PSQI, due to its wide use, allows interpretation of findings in the context of an extensive literature relating sleep to functioning, mental health, and global perception of quality of life (Buysse et al., 1989). Observational studies in outpatient settings (including rheumatology and pain clinics) also describe concomitant self-reported improvement in sleep and symptoms among medical *Cannabis* users (Habib et al., 2021; Aviram et al., 2021). Thus, the results are consistent with the hypothesis that cannabinoid interventions may influence wellbeing through direct and indirect pathways (sleep, relaxation, anxiety reduction, and symptom modulation), beyond the primary control of a target symptom (Zou and Kumar, 2018; Lu and Mackie, 2016; AminiLari et al., 2022; Babson et al., 2017; Amaral et al., 2023; Suraev et al., 2020; Gates et al., 2014).

One of the most relevant findings of the present study was the observed association between longer treatment duration (>3 years) and higher self-reported sleep satisfaction (Table 7). Regarding the broader literature on long-term cannabinoid use, evidence is heterogeneous and often limited by low-certainty studies, with important variation in outcomes, tolerability, and discontinuation rates (Bialas et al., 2022). Guidelines and synthesis reports highlight potential risks of THC, including cognitive and psychiatric effects in certain contexts, especially with chronic high-dose use, early onset, or psychiatric vulnerability, making it essential to avoid overly optimistic interpretations (Bialas et al., 2022; Busse et al., 2021; Nugent et al., 2017). Evidence on the effects of *Cannabis* for chronic pain, a common indication in the present sample, remains limited in strength, with low-certainty evidence suggesting modest analgesic benefit in neuropathic, musculoskeletal, and orofacial pain, and insufficient evidence for other pain types (Bort et al., 2024; Nugent et al., 2017). A comprehensive narrative review including 74 studies and 12,562 patients found that cannabinoids were most effective for chronic secondary musculoskeletal pain, neuropathic pain, and orofacial pain, with treatment initiation recommended at low doses and gradual titration (Bort et al., 2024). At the same time, observational evidence suggests that, under gradual titration and continuous follow-up, some patients maintain subjective benefits over time, with possible stabilization of symptoms and improvement in quality of life (Cahill et al., 2021). It is also fundamental to distinguish contexts of use (supervised medicinal vs. recreational), patient profile, and pharmaceutical form, as these elements may explain discrepancies between clinical findings and studies based on recreational patterns (Bialas et al., 2022; Busse et al., 2021).

Two biases must be explicitly considered to interpret the association observed in this sample between prolonged use and better outcomes. First, since this is a cross-sectional study, there is a risk of reverse causality: patients with better sleep/quality of life may be more likely to maintain treatment for longer periods, while those with poorer response tend to discontinue use earlier (AminiLari et al., 2022; Suraev et al., 2020; Skevington et al., 2004; Bialas et al., 2022). Second, the presence of *survivorship/adherence bias* is plausible, as the “>3 years” group tends to consist of individuals who tolerated the treatment better, perceived benefit, or had better access to follow-up, which may inflate estimates of perceived effectiveness when compared to short-duration groups (Bialas et al., 2022). Therefore, the finding should not be interpreted as proof of superiority of prolonged use per se, but as a signal of perceived effectiveness under specific clinical conditions and with possible selection over time (AminiLari et al., 2022; Suraev et al., 2020; Skevington et al., 2004; Bialas et al., 2022; Davis et al., 2024). Longitudinal evidence suggests a complex and bidirectional interplay between *Cannabis* use and insomnia, wherein *Cannabis* may offer short-term perceived relief while simultaneously contributing to worsening sleep outcomes over time, particularly with increased frequency of use (Davis et al., 2024).

An additional interpretive component when considering the present findings is the possibility of expectation effects in therapies with strong symbolic burden, high patient engagement, and a context of stigma. Evidence on patient experience with medical *Cannabis* suggests that therapeutic expectation, trust in care, and meaning attributed to treatment modulate self-reported outcomes, especially quality of life and sleep, which are highly sensitive to context and perception (Sat et al., 2015; Busse et al., 2021; National Academies of Sciences et al., 2017; Kuhat et al., 2022). Notably, *Cannabis* use for insomnia management has been reported across populations with depression, anxiety, and comorbid conditions, with perceived benefits varying by product form and strain category (Kuhat et al., 2022). Thus, sustained psychosocial effects, combined with clinical follow-up and individualized adjustment, may contribute to perceived improvements even when the isolated pharmacological effect is difficult to separate in observational designs (AminiLari et al., 2022; Gates et al., 2014; Bialas et al., 2022).

In the present study, the finding that absence of adverse effects was associated with a lower probability of high sleep satisfaction is counterintuitive, but plausible under interpretive models centered on effect perception. Reviews indicate that mild adverse events (e.g., drowsiness, xerostomia, dizziness) are relatively frequent and vary according to formulation and dose (Bort et al., 2024; AminiLari et al., 2022; Babson et al., 2017; Amaral et al., 2023; Suraev et al., 2020; Gates et al., 2014; Bialas et al., 2022). In interventions aimed at sleep, mild drowsiness may be perceived as a sign of useful pharmacological effect, while total absence of effects may reflect a subtherapeutic dose, tolerance, underreporting, or lower individual responsiveness, especially in extracts with variable usage (Gates et al., 2014; Bialas et al., 2022; National Academies of Sciences et al., 2017). Therefore, this result should be discussed as a signal of interaction between tolerability, expectation, and perception of benefit, and not as evidence that adverse events are desirable (Bialas et al., 2022; Busse et al., 2021; National Academies of Sciences et al., 2017).

Regarding the associations observed in the present study between racial self-declaration and outcomes, it is recommended to avoid biologizing interpretations and to privilege contextual mechanisms that influence self-reported outcomes. Studies on medical *Cannabis* indicate that stigma, previous experiences with the healthcare system, access barriers, and social insecurity may modulate the experience of treatment and the way benefits are reported (Sat et al., 2015; Busse et al., 2021; National Academies of Sciences et al., 2017). Since quality of life and sleep are outcomes highly sensitive to psychosocial context, differences between groups may reflect variations in therapeutic experience, trust in care, social support, and degree of perceived stigmatization, rather than intrinsic differences in biological response (Skevington et al., 2004; Devlin and Brooks, 2017; Sat et al., 2015; Busse et al., 2021; Arkell et al., 2020). This point reinforces the importance of future studies incorporating socioeconomic variables and stigma/expectation measures to elucidate underlying mechanisms (Sat et al., 2015; Busse et al., 2021; National Academies of Sciences et al., 2017).

In the present study, the absence of a significant association between gender and the outcomes is consistent with part of the literature, which describes greater consistency in differences in use patterns than in perceived efficacy, suggesting heterogeneity according to clinical indication, dose, and sociocultural context (Arkell et al., 2020; Cuttler et al., 2016). Studies also indicate that sex/gender differences may emerge in specific subdomains (e.g., anxiety, consumption patterns, and perceived effects), justifying stratified analyses in larger samples with greater statistical power (Cuttler et al., 2016). Additionally, in diverse clinical populations, gender effects may be mediated by diagnosis, comorbidities, concomitant treatments, and adherence patterns, which is frequently pointed out as a source of heterogeneity in broad reviews (Suraev et al., 2020; Bialas et al., 2022).

Finally, it is important to acknowledge limitations while simultaneously highlighting the contributions of the study. The cross-sectional design, the absence of baseline measures, and the potential selection/response bias limit causal inference and estimation of effect magnitude, and such limitations are widely acknowledged in evidence syntheses on medical *Cannabis* (AminiLari et al., 2022; Suraev et al., 2020; Bialas et al., 2022). Additionally, the ordinal logistic regression models should be interpreted as exploratory, as the sample size (n = 71) and the number of predictors with sparse categories produced parameter instability in specific subcategories, including extreme regression coefficients and non-estimable standard errors consistent with quasi-complete separation. These results are not suitable for direct clinical inference and should be confirmed in larger samples.

The low response rate (71/689; 10.3%) warrants careful consideration, though it is consistent with rates reported in email-based health surveys in real-world clinical populations (Busse et al., 2021; Davis et al., 2024). Online self-administered questionnaires are known to yield substantially lower response rates than paper-based or telephone-administered instruments (Tumyan et al., 2025; Romero et al., 2025), and response rates in this range have been documented even in well-designed randomized studies of survey delivery methods in healthcare settings (Tumyan et al., 2025). In the specific context of medical *Cannabis*, non-response may be further compounded by stigma-related concerns, as a substantial proportion of medical *Cannabis* users conceal their use from healthcare providers due to fear of disapproval or legal concerns (Kongsved et al., 2007). Nonetheless, selection bias remains a concern: respondents may systematically differ from non-respondents in terms of treatment adherence, satisfaction with outcomes, and capacity to complete a lengthy self-administered instrument combining two validated multidomain instruments, potentially overestimating perceived effectiveness and underrepresenting patients with poor outcomes or treatment discontinuation. Furthermore, data were collected via a self-administered online questionnaire, which carries inherent response biases: interpretation bias may arise from individual variability in how participants understand questionnaire items; memory bias is inherent to retrospective self-report instruments such as the PSQI; expectation bias is particularly relevant in the context of medical *Cannabis*, where strong prior beliefs about therapeutic benefits may inflate self-reported outcomes; and social desirability bias may lead participants to report more favorable outcomes than actually experienced. These limitations are particularly pertinent given that the primary outcomes (quality of life and sleep satisfaction) are highly subjective and context-sensitive.

The need for variable grouping may reduce granularity, but it is a defensible strategy for statistical stability in moderate and heterogeneous samples. The clinical heterogeneity of the sample, encompassing adults, children, adolescents, and patients with psychiatric, neurological, pain-related, and other conditions analyzed jointly, represents an important contextual consideration. The comparative analyses conducted were explicitly exploratory in nature, designed to identify preliminary signals and generate hypotheses rather than to establish definitive subgroup-specific conclusions. Given the sample size and the distribution of responses across clinical subgroups, drawing direct or conclusive comparisons between groups was neither statistically appropriate nor the primary objective of the study. This approach is consistent with the broader aim of characterizing real-world patterns of *Cannabis* oil use in a heterogeneous clinical population, reflecting the complexity of actual clinical practice. Subgroup-specific confirmatory analyses would require substantially larger and more homogeneous samples, and are strongly recommended as a priority for future longitudinal research. On the other hand, clinical heterogeneity may be interpreted as an external validity strength, as it mirrors the reality of services that follow patients with multiple indications and individualized therapeutic regimens, an aspect frequently absent in highly selected randomized clinical trials (AminiLari et al., 2022; Gates et al., 2014; Aran et al., 2021; Bialas et al., 2022). Thus, the findings contribute by describing the performance of a cannabinoid intervention with a focus on PROMs, offering hypotheses for future longitudinal investigations with baseline assessment, follow-up, and better control of confounders, in addition to more detailed product characterization, including phytochemical profile.

In summary, the results suggest that, in supervised clinical practice, patients using full-spectrum THC and CBD rich oil reported favorable perceived quality of life and sleep satisfaction. However, given the cross-sectional design and the absence of pre-treatment assessment, these findings reflect self-reported perceptions rather than evidence of treatment efficacy, and causal interpretation is not warranted. Future longitudinal studies, with pre-treatment assessment, systematic follow-up, and more detailed product characterization, are needed to clarify mechanisms, responding subgroups, and the risk-benefit balance of prolonged use, especially in products with higher THC content.

## Data Availability

The original contributions presented in the study are included in the article/supplementary material, further inquiries can be directed to the corresponding author.
